# Speeding up enzyme engineering computationally

**DOI:** 10.1107/S2052252516019692

**Published:** 2016-12-24

**Authors:** Nigel S. Scrutton

**Affiliations:** a Synthetic Biology Research Centre for Fine and Speciality Chemicals, Manchester Institute of Biotechnology and School of Chemistry, The University of Manchester, Manchester Institute of Biotechnology, 131 Princess Street, Manchester M1 7DN, UK

**Keywords:** *CADEE*, computational directed evolution, computational enzyme design, distributed computing, empirical valence bond, triosephosphate isomerase

## Abstract

Can *in silico* engineering speed up the delivery of biocatalysts for the burgeoning bioeconomy? In this issue, Kamerlin and coworkers introduce *CADEE* [Amrein *et al.* (2017), *IUCrJ*, **4**, 50–64] – a framework for Computer-Aided Directed Evolution of Enzymes – that promises to lessen the burden on ‘wet lab’ enzymologists when optimizing biocatalysts using laboratory-based directed evolution methods.

These are exciting times for the enzyme engineer. The emergence of the bioeconomy has defined new challenges to extend the chemistry of natural enzymes and to engineer new properties necessary for industrial-scale chemicals production. When incorporated into existing synthetic manufacturing routes, biocatalysts can drive improvements in product stereochemistry, chemical diversity, enantiopurity and functionality, as well as process sustainability. This emphasizes the overall benefits of using enzyme catalysts in industrial manufacture. As we transition from a petrochemical to a more sustainable, green bioeconomy we must ensure that our ability to design and engineer biocatalysts does not become a bottleneck in the industrialization of biology. This is a grand challenge for the enzyme engineer and one that needs to be addressed with some urgency.

So how do we speed up enzyme engineering to provide much-needed new biocatalysts for the bioeconomy and biotechnological applications? Traditional ‘rational’ protein engineering has been bedevilled with problems stemming, in the main, from the complexity of dynamic protein structures and often a lack of in-depth understanding of the ‘catalytic effect’. Informed by structure, early days witnessed attempts to redesign enzyme specificity towards new (often chemically related) substrates, but with mixed success. Typically slow and painstaking rounds of optimization were needed to reach target functions (*e.g.* substrate specificity, product enantiopurity) or process properties (*e.g.* temperature stability, solvent compatibility). This has led to the emergence of improved experimental methods for directed evolution of enzymes in biocatalyst engineering workflows (Toogood & Scrutton, 2013 ▸; Currin *et al.*, 2015 ▸). The fundamental challenge however remains, *i.e.* the need to explore a vast amount of sequence space to optimize a biocatalyst towards the target property or function. The burden of ‘the numbers game’ can be lessened – but only to some extent – by employing semi-random approaches in gene library synthesis, or through the use of SMART libraries, rather than relying on the more traditional approaches of random mutagenesis or recombination. Advances in high-throughput screening (*e.g.* using fluorescence-activated cell sorting, phage display or **in vitro** compartmentalization) can meet the need to screen large numbers of variants. This is especially powerful when coupled to new methods of library generation where it is possible to control the extent and nature of substitutions in a gene library using synthetic biology methods (Currin *et al.*, 2015 ▸).

Notwithstanding the above, experimental workflows for enzyme engineering and directed evolution have placed great demands on person time and laboratory resources, even with laboratory automation. This is clearly not sustainable when new biocatalysts are urgently needed to support the industrialization of biology. The field is crying out for more predictive design based on an iterative Design, Build, Test, Learn (D/B/T/L) strategy that involves a cyclic process of developing an initial enzyme prototype, testing the prototype, analyzing its performance and learning what works/what does not work, prior to moving on in an informed way to the next round of the cycle. The *de novo* enzyme design field has been leading the way in generating prototype protein scaffolds to generate new catalysts using *in silico* approaches. Missing, however, has been a rapid ‘Test’ computational platform to embed in enzyme engineering workflows that allows one to rapidly (*i.e.* at low computational cost) assess *in silico* the outcome of multiple amino acid substitutions prior to building and testing a limited set of these variants in the ‘wet-lab’.

In this issue of **IUCrJ**, Kamerlin and co-workers (Amrein *et al.*, 2017 ▸) now describe a computational framework for the computer-aided directed evolution of enzymes (*CADEE*) that can rapidly screen the quantitative effects of thousands of amino-acid substitutions on catalytic function and at relatively low computational cost. Their method uses the empirical valence-bond (EVB) approach (Warshel & Weiss, 1980 ▸; Hwang *et al.*, 1988 ▸) to predict activation barriers for chemical reactions across many enzyme variants generated by *in silico* mutagenesis. By avoiding high-level quantum-mechanical approaches (which are computationally very expensive), or semi-empirical QM/MM approaches (which are of limited accuracy), Kamerlin and coworkers argue that the EVB approach (which is a valence-bond classical approach) is ideal for assessing rapidly and with quantitative accuracy the effects of multiple amino-acid substitutions. The EVB approach therefore enables rapid *in silico* analysis of thousands of computationally generated enzyme variants with reasonable computational cost.

The *CADEE* framework is available as a Python 2.7 application and is available for download to the wider community. Although EVB approaches have been used previously in limited screening studies, *CADEE* for the first time provides a semi-automated framework for performing large numbers of EVB calculations, as would be typical for *in silico* directed evolution experiments. As with all computational approaches there are caveats: the authors emphasize that ‘*CADEE requires a well calibrated reference state, ideally benchmarked against the effect of a number of experimentally characterized amino acid substitutions*’. This is needed to generate a high-quality EVB force field to perform *in silico* simulations requiring rigorous parameterization of EVB potentials.

*CADEE* is a welcome computational platform tool that should be embedded into Design, Build, Test, Learn (D/B/T/L) pipelines/strategies for enzyme engineering (see Fig. 1 ▸). In offering a framework for semi-automated *in silico* directed evolution of enzymes *CADEE* should contribute to the need for speeding up design and *in silico* testing of enzyme variants and the streamlining of ‘wet lab’ resources required to generate next generation biocatalysts. As such *CADEE* is an important computational tool for the enzyme engineer. It adds to the predictable and rapid enzyme redesign toolbox, which is fundamentally important to growth of the bioeconomy.

## Figures and Tables

**Figure 1:**
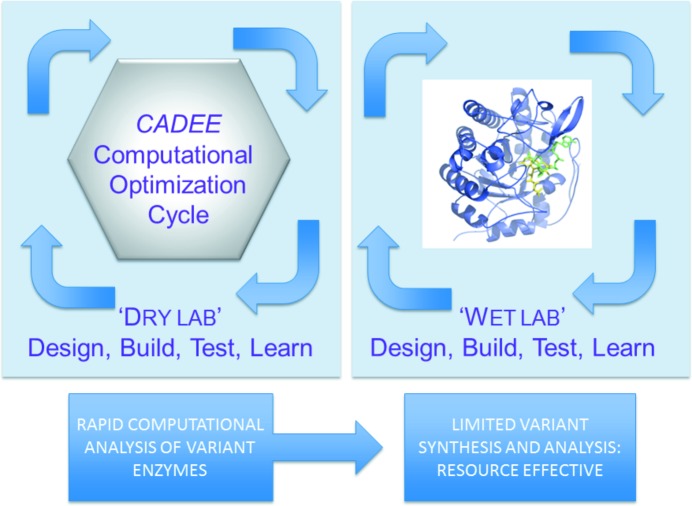
*CADEE* and Design, Build, Test, Learn iterative cycles. *CADEE* and other predictive computational tools in the enzyme engineering ‘dry lab’ lessens burden on resources and experimental effort in the design, build, test and learn cycles of the ’wet lab’.

## References

[bb1] Amrein, B. A., Steffen-Munsberg, F., Szeler, I., Purg, M., Kulkarni, Y. & Kamerlin, S. C. L. (2017). *IUCrJ*, **4**, 50–64. 10.1107/S2052252516018017 PMC5331465 28250941

[bb2] Currin, A., Swainston, N., Day, P. J. & Kell, D. B. (2015). *Chem. Soc. Rev.* **44**, 1172–1239. 10.1039/c4cs00351a PMC4349129 25503938

[bb3] Hwang, J. K., King, G., Creighton, S. & Warshel, A. (1988). *J. Am. Chem. Soc.* **110**, 5297–5311.

[bb4] Toogood, H. S. & Scrutton, N. S. (2013). *Catal. Sci. Technol.* **3**, 2182–2194.

[bb5] Warshel, A. & Weiss, R. M. (1980). *J. Am. Chem. Soc.* **102**, 6218–6226.

